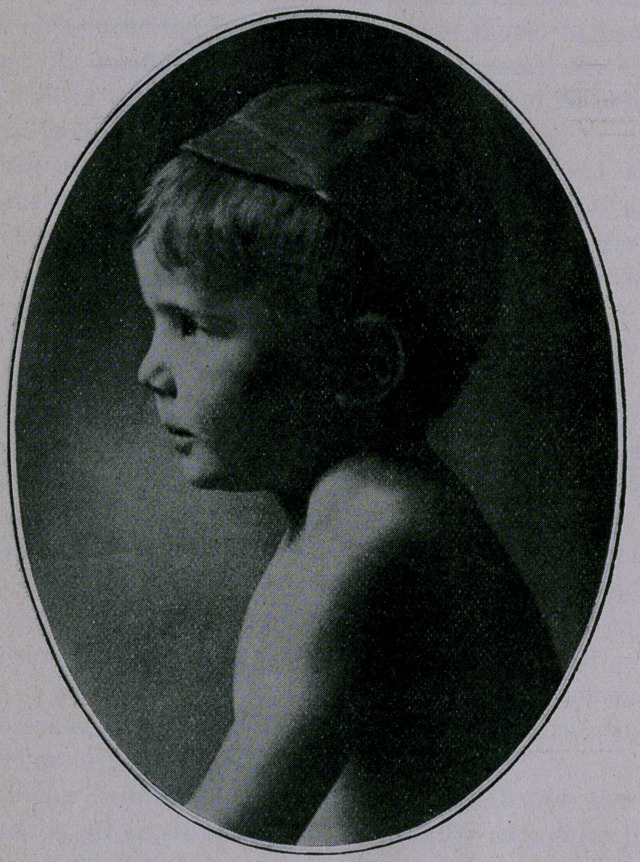# Lymphatic Cyst of the Neck—Report of a Case

**Published:** 1908-12

**Authors:** C. S. Venable

**Affiliations:** San Antonio


For Texas Medical Journal.

### Lymphatic Cyst of the Neck—Report of a Case.

BY DR. C. S. VENABLE, SAN ANTONIO.

A child, 5 years of age, was brought to me by its mother complaining of a "lump" on the side of his neck, which the accompanying photographs show better than I can describe.

The family history was good, there being no tuberculosis, syphilis or malignancy on either side. He has three brothers and two sisters in perfect health. His past history was that he had had measles a year and a half previously, followed by a severe attack of bronchitis, at which time his mother first noticed a small swelling on the left side of his neck. This gradually enlarged, and in nine months had reached the size of a pigeon egg. Here he had a second attack of bronchitis, and in the succeeding eight months the growth became as large as a hen egg. He has always been well nourished, now weighing 46 pounds, and suffering no inconvenience other than his collar is uncomfortable. Upon examination a mass was felt under the trapezius on the left side, extending backward beyond its posterior border, and forward beneath the posterior border of the sterno-mastoid muscle at its middle third. The overlying skin was freely movable while the tumor was immovable, fluctuated and elicited no pain or tenderness on manipulation, or any tendency to disappear on pressure. It exhibited no bruit or breath sounds on auscultation, and there was no other lymphatic or associated inflammatory involvement. The chest was well formed, expansion good and normal vesicular resonance with clear breath sounds over the entirety of both lungs, there being no latent evidence of the previous bronchitis. No puncture for examination of the sac contents was made, but complete extirpation advised, which was consented to.

On operation I found the small end to the front, snugly embedded under the sterno-mastoid, though not quite extending to the vessels of the neck; above a thick band of fibers bound it to the inner face of the tip of the mastoid process, while behind it was held by a thick layer of connective tissue fibers to the transverse processes of the last cervical and first dorsal vertebræ. Throughout the dissection there was found no remains at an early connection with the air passages, the sac being entirely occluded and removed intact, it being a little larger than a turkey egg.

The pathological report was that it was a cyst of lymphatic origin presenting a smooth surface consisting of a connective tissue capsule with an endothelial lining, was monolocular and contained a thin, clear, mucous secretion. There was no evidence of the remains of a bronchial cyst, or any of the histological or pathological elements characteristic of a dermoid or of a tuberculous gland having undergone secondary cystic degeneration. The wound healed by first intention and two years has elapsed with no sign of recurrence or later lymphatic involvement.

I wish to make acknowledgment of the assistance rendered by Dr. C. H. Bunting, then Professor of Pathology in the University of Virginia, now of Wisconsin, in reaching a conclusion as to the origin of this cyst, which was impossible from the clinical aspect alone.

## Figures and Tables

**Figure f1:**
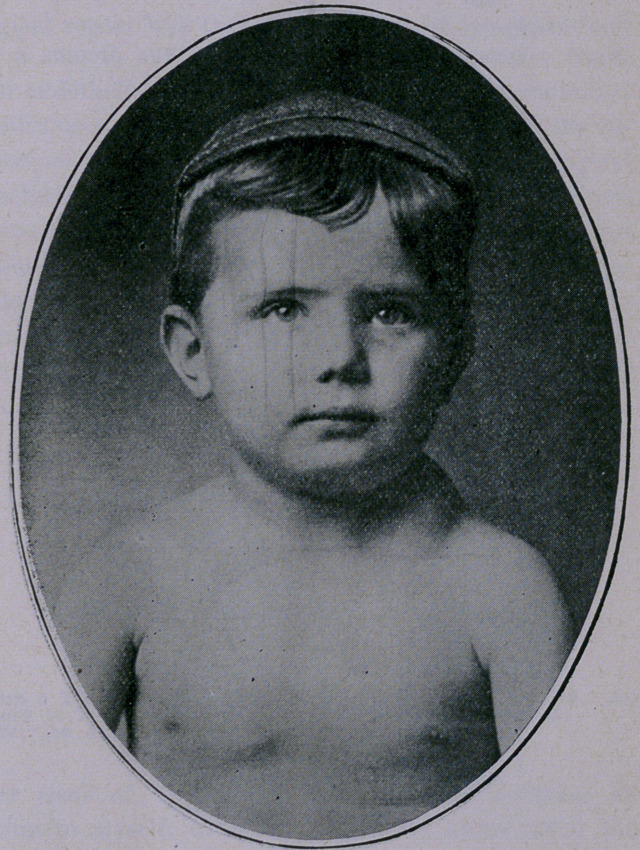


**Figure f2:**